# Complete chloroplast genome sequence and phylogenetic analysis of winter oil rapeseed (*Brassica rapa* L.)

**DOI:** 10.1080/23802359.2020.1860697

**Published:** 2021-03-11

**Authors:** Jun Yan Wu, Xue Cai Ma, Li Ma, Yan Fang, Ya Hong Zhang, Li Jun Liu, Xue Cai Li, Rui Zeng, Wan Cang Sun

**Affiliations:** a College of Agronomy, Gansu Agricultural University/Rapeseed Engineering Research Center of Gansu Province, Lanzhou, China; b Tianshui Institute of Agricultural Sciences, Tianshui Gansu, China

**Keywords:** Winter oil rapeseed, chloroplast genome, SSRs, phylogeny

## Abstract

Winter oil rapeseed ‘18 R-1’ (*Brassica rapa* L.) is a new variety that can survive in northern China where the extreme low temperature is −20 °C to −32 °C. It is different from traditional *B. rapa* and *Brassica napus*. In this study, the complete chloroplast (cp) genome of ‘18 R-1’ was sequenced and analyzed to assess the genetic relationship. The size of cp genome is 153,494 bp, including one large single copy (LSC) region of 83,280 bp and one small single copy (SSC) region of 17,776 bp, separated by two inverted repeat (IR) regions of 26,219 bp. The GC content of the whole genome is 36.35%, while those of LSC, SSC, and IR are 34.12%, 29.20%, and 42.32%, respectively. The cp genome encodes 132 genes, including 87 protein-coding genes, eight rRNA genes, and 37 tRNA genes. In repeat structure analysis, 288 simple sequence repeats (SSRs) were identified. Cp genome of ‘18 R-1’ was closely related to *Brassica chinensis*, *B. rapa* and *Brassica pekinesis*.

## Introduction

Winter oil rapeseed (*Brassica rapa* L.) is a new cultivar used as oil crop in northern China. It can survive in fields where the extreme low temperature is −20 to −32 °C in winter. It makes northern China grow winter rapeseed now where is spring rapeseed zone before (Wancang et al. 2010; Dongmei et al. 2014). Growing winter rapeseed has many advantages in northern China. Firstly, winter rapeseed sows in mid-August, and turns green in late March next year, harvests in early June which is one and a half months earlier than spring crops. So after harvests, it is possible for a succeeding crop such as maize, potato, millet, corn, buckwheat, vegetables and others (Sun et al. 2016). It can make full use of heat and light of this area and change the traditional 1-year-one-ripe pattern to a 2-year-three ripe pattern (Wang et al. 2009). In this way, it can avoid spring farming in this area and result in increasing land cover during winter. So winter rapeseed is a cover crop in winter and it will reduce soil surface dust (Xuefang et al. 2009).

Chloroplast (cp) is common in plant and other organism. Because it is simple, conservative and rearranged, it is mainly used to analyze the origin and evolution of species (Szymon et al. 2016). In our research, we find that winter rapeseed (*B. rapa*) is different from *B. napus* and *B. rapa* cultivars. It has strong cold tolerance and low growing point. So in this study, we used a winter oil rapeseed variety, ‘18 R-1’ and constructed its whole cp genome. We compared the cp genome with other members of *Brassica* to make sure its genetic evolutionary relationship. It is expected that the results will provide a theoretical basis for the determination of phylogenetic status and future breeding research.

## Materials and methods

### Sampling, DNA extraction, sequencing, and assembly

The experiments were set up in Gansu Agricultural University, China (N. 36.05°, E. 103.87°). ‘18 R-1’ seeds were sowed in pot. Fresh leaves were collected at five-leaf-stage and were frozen in liquid nitrogen immediately then stored at −80 °C until analysis. Genomic DNA was extracted by the modified method CTAB (Li et al. 2019). After testing qualified, genomic DNA samples were broken into fragments with the mechanical interrupt method (ultrasonic). Then fragment purification, terminal repair, the addition of 3’terminal A and connection of sequencing connector were performed for fragmented DNA. The fragment size was selected by agarose gel electrophoresis, and the sequencing library was formed by PCR amplification. The library was inspected first, then the qualified library shall be sequenced and sequencing reading length was PE150. Sequencing was performed with an Illumina Hiseq 2500 platform (Nanjing, China, N. 31.14°, E. 118.22°), yielding at least 11.02 GB of clean base. All of the raw reads were trimmed by Fastqc. The core module was assembled using SPAdes (Bankevich et al. 2012) software to assemble the chloroplast genome, independent of the reference genome.

### Annotation and analysis of the cpDNA sequences

CpGAVAS was used to annotate the sequences. DOGMA (http://dogma.ccbb.utexas.edu/) and BLAST were used to check the results of the annotation (Liu et al. 2012; Wyman et al. 2004). The circular gene map of 18 R-1 was drawn using the OGDRAWv1.2 program (Lohse et al. 2007). An analysis of variation in synonymous codon usage, relative synonymous codon usage values (RSCU), codon usage, and GC content of the complete plastid genomes and commonly analyzed CDS was conducted. CpSSR analysis was performed using the MISA (Song et al. 2019).

### Genome comparison

The mVISTA (Mayor et al. 2000) program was applied to compare the complete cp genome of ‘18 R-1’ to the other published cp genomes of its related species.

### Phylogenetic analysis

It used genome-wide analysis by setting the same starting points for ring sequences. Multiple sequence alignment was performed with MAFFT software (v7.427, auto mode). Sequence alignment data were trimmed with trimAl (v1.4.rev15). Then using RAxML v8.2.10 software (https://cme.h-its.org/exelixis/software.html) and GTRGAMMA model, we built maximum likelihood evolutionary tree with rapid Bootstrap analysis (Bootstrap = 1000). Phylogenetic tree was constructed using 25 cp genome of the Cruciferae species sequences from the NCBI organelle genome and nucleotide resources database (Katoh et al. 2005; Lam-Tung et al. 2015; Huelsenbeck and Ronquist 2001; Xiayu et al. 2019).

## Results and discussion

### Cp genome size

**Figure 1.**
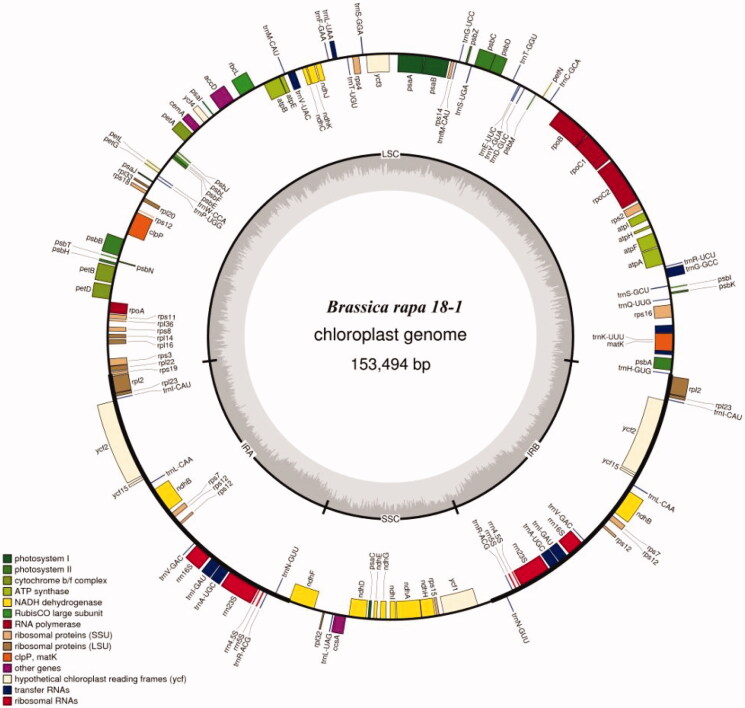
Cp genome map of 18 R-1. Genes inside the circle are transcribed clockwise, and those outside are transcribed counterclockwise. Genes of different functions are color-coded. The darker gray in the inner circle shows the G + C content, while the lighter gray shows the A + T content.

The accession number of complete cp genome on Genebank is MT726210 (https://www.ncbi.nlm.nih.gov/nuccore/MT726210). The size of ‘18 R-1’ cp genome is 153,494 bp which has 132 genes including 37 tRNAs, eight rRNAs, and 87 mRNAs (Figure 1; Table 1). Most genes have only one copy, while 19 genes (ndhB, rpl2, rpl23, rps12, rps7, rrn16S, rrn23S, rrn4.5S, rrn5S, trnA-UGC, trnI-CAU, trnI-GAU, trnL-CAA, trnN-GUU, trnR-ACG, trnV-GAC, ycf1, ycf15, ycf2) with two copies. The cp genome displayed a typical quadripartite structure, including a pair of IR (IRA and IRB, 26,219 bp) separated by the large single copy (LSC; 83,280 bp) and small single copy (SSC; 17,776 bp) regions (Figure 1). The DNA GC contents of LSC, SSC, IR, and the whole genome are 34.12%, 29.20%, 42.32%, and 36.35%, respectively. It is obvious that GC content of the IR region is higher than that of other regions. This phenomenon is very common in other plants (Nguyen et al. 2015). GC skewness has been shown to be an indicator of DNA lead chains, lag chains, replication origin, and replication terminals (Dan et al. 2019; Yan et al. 2018).

**Table 1.** List of genes annotated in the cp genomes of winter rapeseed.

Function	Genes
RNAs, transfer	*trnH-GUG, trnS-GCU, trnG-GCC*^a^*, trnC-GCA, trnD-GUC, trnY-GUA, trnT-GGU, trnF-GAA, trnS-GGA, trnI-GAU*^a^*, trnN-GUU, trnV-GAC, trnG-UCC, trnE-UUC, trnK-UUU*^a^*, trnQ-UUG, trnR-UCU, trnS-UGA, trnT-UGU, trnL-UAA*^a^*, trnS-UAG, trnV-UAC*^a^*, trnL-UAG, trnA-UGC*^a^*, trnA-UCG, trnP-UGG, trnfM-CAU, trnM-CAU, trnI-CAU, trnW-CCA, trnA-CGU, trnL-CAA, trnL-CAA, trnR-ACG*
RNAs, ribosomal	*rrn16, rrn23, rrn4.5, rrn5*
Transcription and splicing	*rpoA, rpoB, rpoC1*^a^*, rpoC2*
Translation, ribosomal proteins	*rps12*^b^*, rpl36, rpl32*
Small subunit	*rps2, rps4, rps14, rps133, rps18, rps11, rps8, rps3, rps7, rps15*
Large subunit	*rpl20, rpl12, rpl14, rpl16, rpl22, rps19, rpl2*^a^*, rpl23*
ATP synthase	*atpA, atpF*^a^*, atpH, atpI, atpB, atpE,*
Photosystem I	*psaA, psaB, psaC, psaI, psaJ, ycf1, ycf3*^b^*, ycf4*
Photosystem II	*psbA, psbB, psdC, psbD, psbE, psbF, psbH, psbI, psbJ, psbK, psbL, psbM, psbN, psbZ, psbT*
Calvin cycle	*rbcL*
Cytochrome complex	*petA, petL, petB*^a^*, petD*^a^*, ccsA*
NADH dehydrogenase	*ndhJ, ndhK, ndhC, ndhB*^a^*, ndhF, ndhE, ndhG, ndhA*^a^*, ndhH, ndhD*
Others	*cemA, clpP, clpP*^b^*, ycf2, ycf15*^a^*, accD*

^a^Genes containing one intron; ^b^Genes containing two introns.

Seventeen intron-containing genes were found in the cp genome including 12 genes with 1 intron and 3 genes with 2 introns (Table 1). Introns also vary in size. The trnK-UUU has longest intron (2561 bp) and trnL-UAA has the shortest (313 bp).

### RSCU analysis

RSCU is relative synonymous codon usage. Because of the degeneracy of codons, each amino acid corresponds to at least 1 codon and at most 6 codons. The utilization rate of genomic codon varies greatly among different species and organisms. RSCU is thought to be the result of natural selection, mutation and genetic drift.

**Figure 2.**
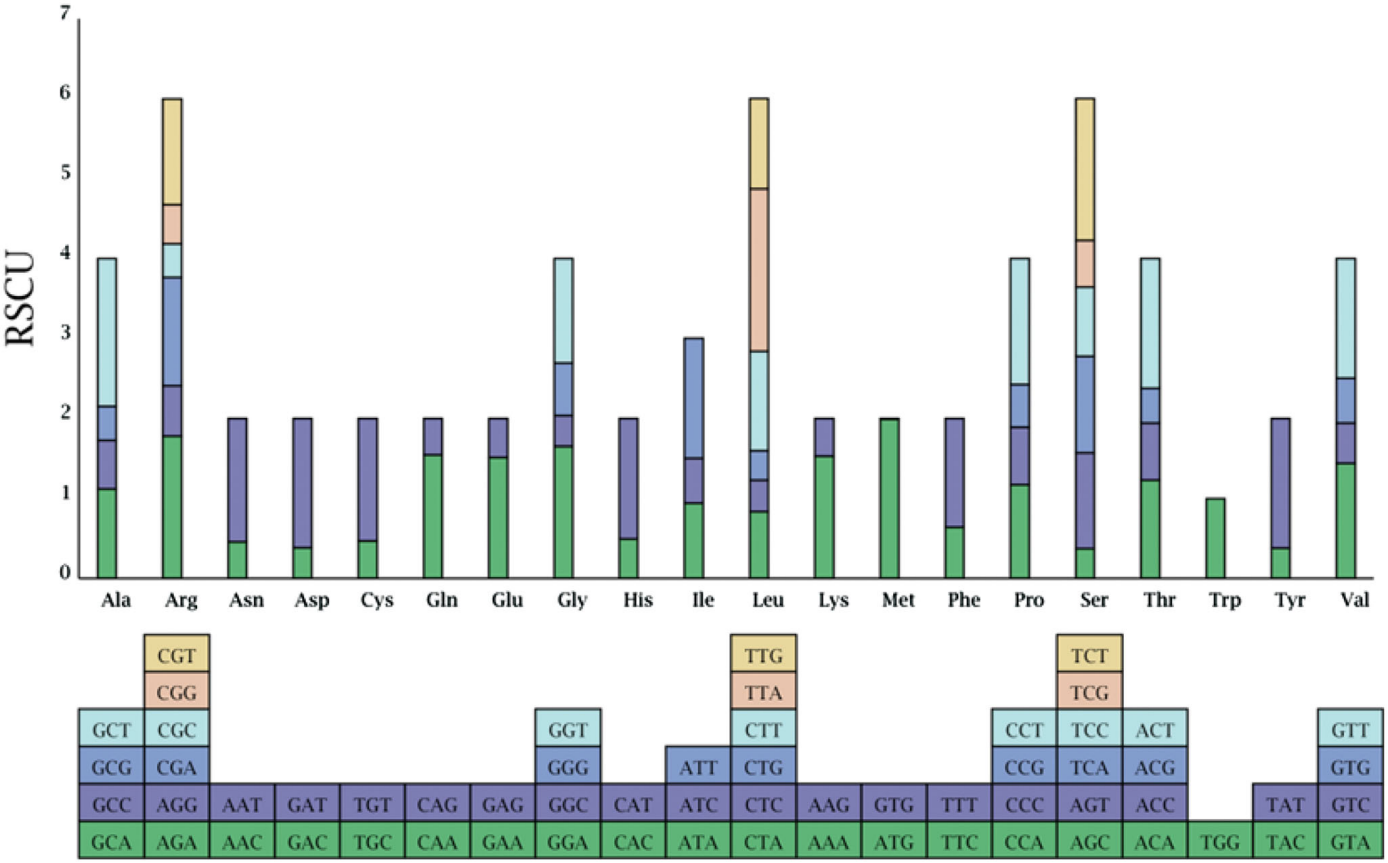
RSCU distribution.

Regardless of termination codon, UUA encoding ‘Leu’ was the most used codon, while GUG encoding ‘Met’ was the fewest used codon (Table 2; Figure 2). There are 30 codons with RSCU greater than 1, in which the third base are all ending in A/U. It indicated that winter rapeseed preferred to use the codon ending in A/U in the third base. There is only one codon, UGG, which RSCU is 1.

**Table 2.** Codon preference analysis statistics.

Amino acid	Symbol	Codon	No.	RSCU
*	Ter	UAA	52	1.7931
	Ter	UAG	23	0.7932
	Ter	UGA	12	0.4137
A	Ala	GCA	381	1.1232
	Ala	GCC	205	0.6044
	Ala	GCG	143	0.4216
	Ala	GCU	628	1.8512
C	Cys	UGC	74	0.4684
	Cys	UGU	242	1.5316
D	Asp	GAC	197	0.3870
	Asp	GAU	821	1.6130
E	Glu	GAA	1014	1.5146
	Glu	GAG	325	0.4854
F	Phe	UUC	498	0.6422
	Phe	UUU	1053	1.3578
G	Gly	GGA	718	1.6524
	Gly	GGC	167	0.3844
	Gly	GGG	285	0.6560
	Gly	GGU	568	1.3072
H	His	CAC	146	0.4940
	His	CAU	445	1.5060
I	Ile	AUA	702	0.9435
	Ile	AUC	419	0.5631
	Ile	AUU	1111	1.4934
S	Ser	AGC	125	0.3750
	Ser	AGU	399	1.1964
	Ser	UCA	402	1.2054
	Ser	UCC	289	0.8664
	Ser	UCG	194	0.5820
	Ser	UCU	592	1.7754
M	Met	AUG	588	1.9898
	Met	GUG	3	0.0102
T	Thr	ACA	411	1.2304
	Thr	ACC	237	0.7096
	Thr	ACG	146	0.4372
	Thr	ACU	542	1.6228
K	Lys	AAA	1110	1.5290
	Lys	AAG	342	0.4710
L	Leu	CUA	386	0.8364
	Leu	CUC	183	0.3966
	Leu	CUG	168	0.3636
	Leu	CUU	573	1.2414
	Leu	UUA	939	2.0340
	Leu	UUG	521	1.1286
N	Asn	AAC	290	0.4574
	Asn	AAU	978	1.5426
P	Pro	CCA	306	1.1712
	Pro	CCC	188	0.7196
	Pro	CCG	140	0.5360
	Pro	CCU	411	1.5732
Q	Gln	CAA	713	1.5450
	Gln	CAG	210	0.4550
R	Arg	AGA	454	1.7790
	Arg	AGG	160	0.6270
	Arg	CGA	346	1.3560
	Arg	CGC	108	0.4230
	Arg	CGG	124	0.4860
	Arg	CGU	339	1.3284
V	Val	GUA	501	1.4408
	Val	GUC	174	0.5004
	Val	GUG	196	0.5636
	Val	GUU	520	1.4952
W	Trp	UGG	441	1.0000
Y	Tyr	UAC	182	0.3816
	Tyr	UAU	772	1.6184

*Note*: ‘*’ means stop codon.

### Repeat sequence and SSR analysis

By the REPuter analysis, there are 37 repeat sequences in the cp genome (Table 3). Except for one repeat with the length of 26,219 bp, the others are 30 bp to 58 bp. Most of the repeats are located in LSC region. Palindrome repeats are 18 while forward repeats are 14, and reverse and complement repeats are 3 and 2, respectively.

**Table 3.** Repetitive sequences identified in the cp genome.

No.	Size (bp)	Type	Repeat I start	Gene	Region	Repeat II start	Gene	Region
1	58	F	47,009	trnF-GAA	LSC	47,063	trnF-GAA	LSC
2	46	F	37,864	psaB	LSC	40,088	psaA	LSC
3	45	P	75,812	petD	LSC	75,812	petD	LSC
4	44	P	73,309	IGS	LSC	73,309	IGS	LSC
5	43	F	37,843	psaB	LSC	40,067	psaA	LSC
6	42	F	27,344	IGS	LSC	27,364	IGS	LSC
7	40	P	28,246	IGS	LSC	28,246	IGS	LSC
8	37	F	97,927	IGS	IRb	119,493	ndhA	SSC
9	37	P	119,493	ndhA	SSC	138,810	IGS	IRa
10	37	C	7944	IGS	LSC	35,443	IGS	LSC
11	36	P	9237	IGS	LSC	9237	IGS	LSC
12	35	R	4588	IGS	LSC	4588	IGS	LSC
13	34	F	106,834	IGS	IRb	106,866	IGS	IRb
14	34	P	106,834	IGS	IRb	129,874	IGS	IRa
15	34	P	106,866	IGS	IRb	129,906	IGS	IRa
16	34	F	129,874	IGS	IRa	129,906	IGS	IRa
17	33	P	172	IGS	LSC	175	IGS	LSC
18	32	F	7949	IGS	LSC	35,447	IGS	LSC
19	32	F	88,213	ycf2	IRb	88,234	ycf2	IRb
20	32	P	88,213	ycf2	IRb	148,508	ycf2	IRa
21	32	P	88,234	ycf2	IRb	148,529	ycf2	IRa
22	32	F	148,508	ycf2	IRa	148,529	ycf2	IRa
23	31	F	7635	trnS-GCU;	LSC	34,497	trnS-UGA	LSC
24	31	R	172	IGS	LSC	34,592	trnS-UGA	LSC
25	31	R	12,208	atpF	LSC	75,812	petD	LSC
26	30	F	47,037	trnF-GAA	LSC	47,091	IGS	LSC
27	30	P	7636	trnS-GCU	LSC	44,059	trnS-GGA	LSC
28	30	F	42,956	ycf3	LSC	97,936	IGS	IRb
29	30	P	42,956	ycf3	LSC	138,808	IGS	IRa
30	30	P	61,677	IGS	LSC	61,677	IGS	LSC
31	30	P	64,761	IGS	LSC	64,761	IGS	LSC
32	30	P	122,764	IGS	SSC	123,309	ycf1	SSC
33	30	F	3758	trnK-UUU	LSC	6274	IGS	LSC
34	30	P	34,498	trnS-UGA	LSC	44,059	trnS-GGA	LSC
35	30	P	34,566	trnS-UGA	LSC	43,997	trnS-GGA	LSC
36	30	C	7937	IGS	LSC	35,447	IGS	LSC
37	26,219	P	83,280		IR	127,275		IR

*Note*: F: forward; P: palindromic; R: reverse; C: complement; IGS: intergenic region.

**Figure 3.**
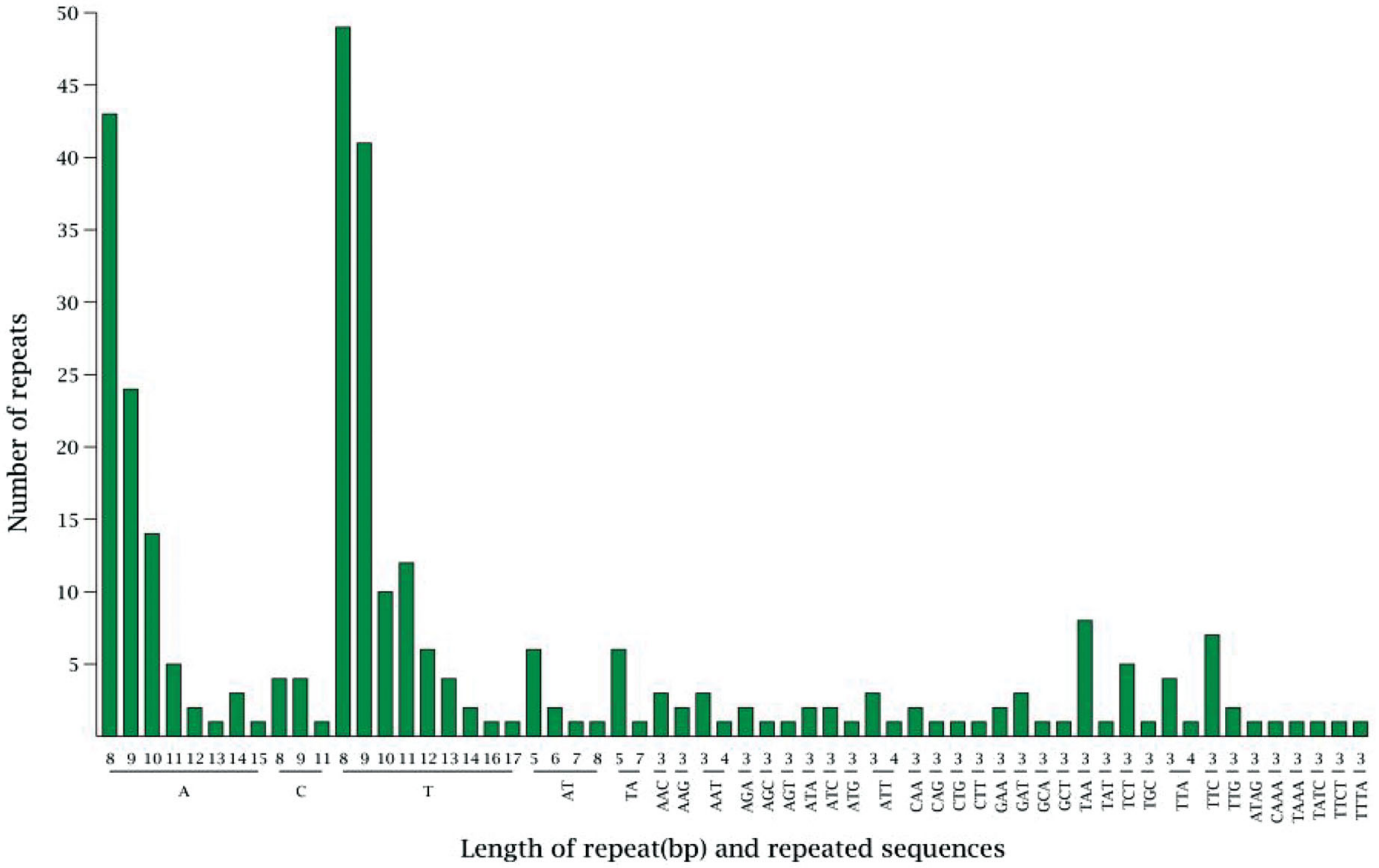
Length of repeat (bp) and repeated sequence.

**Figure 4.**
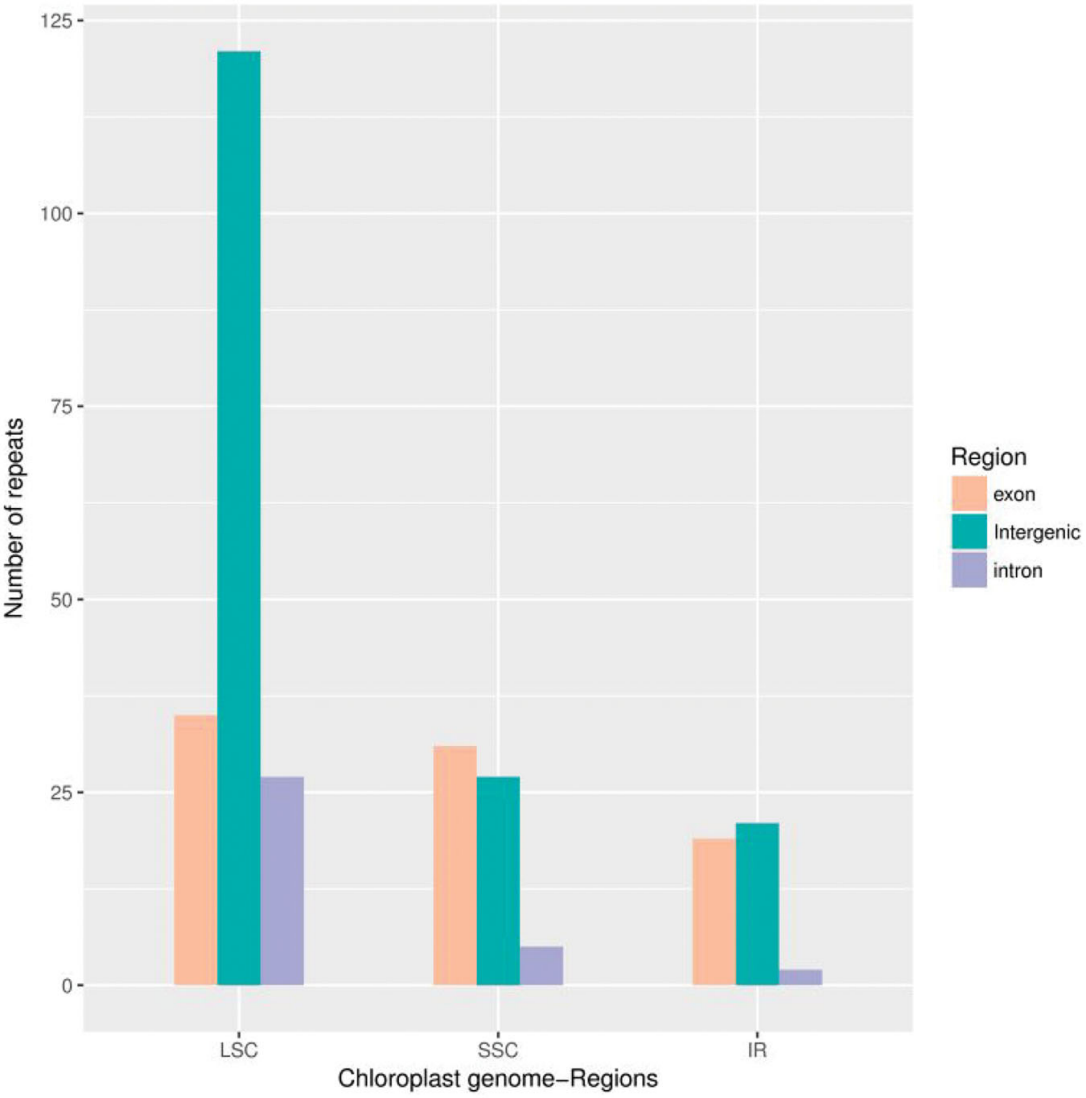
SSR sequences of cp genome.

The cp genome has 288 SSRs, including 228 mononucleotide repeats which are mainly A and T, 17 dinucleotide repeats, 63 trinucleotide repeats and 6 tetranucleotide repeats (Figure 3). From the location of SSR distribution, the vast majority (63.50%) is located in LSC region, and 21.90% located in SSC region and 14.60% in IR region (Figure 4). The SSRs of tandem guanine (G) and cytosine (C) is fewer which means it has strong A and T bias. Most SSRs are distributed in intergenomic region, followed by exon region, and intron region was the least. These repeated sequences can be applied to the development of molecular markers and provide guidance for the evolutionary study of winter rapeseed.

### IR scope analysis

**Figure 5.**
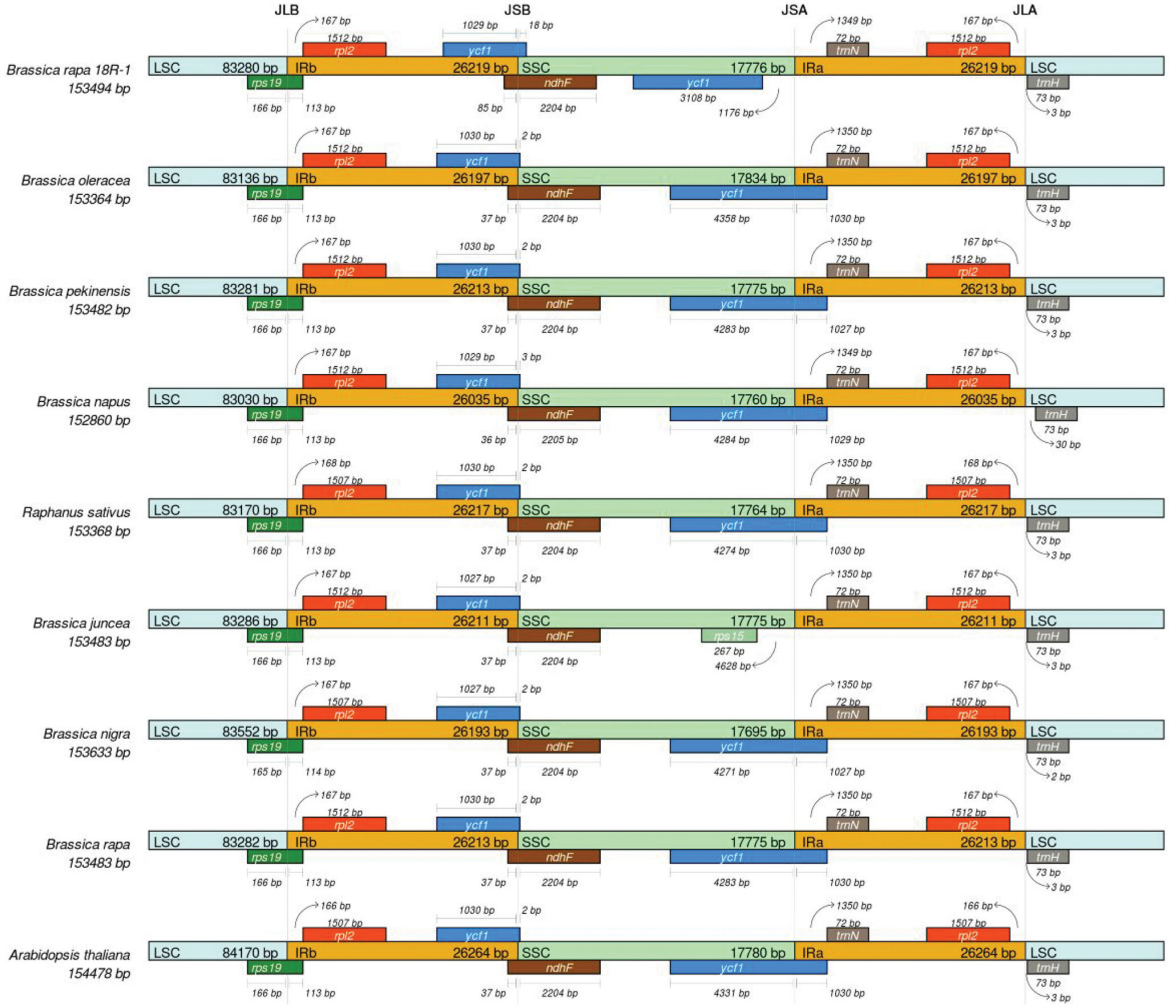
Analysis of cp IR scope change.

Cp genomes of other eight *Cruciferous* species were selected for comparative analysis of LSC/IRs and SSC/IRs boundaries with 18 R-1. The LSC/IRb boundary of 9 species located in the coding region of *rps19*, which spans two regions and is 166 bp at LSC region while 113 bp at IRb region. It is reported that LSC/IRb boundary is stable in many species (Zhao et al. 2019). In most species, IRb/SSC boundary lies in the overlap region between *ycf1* gene and *ndhF* gene (Zhao et al. 2019). In 9 *Cruciferous* species the IRb/SSC boundary is *ycf1* and *ndhF* too. At SSC/IRa boundary, *ycf1* straddles the edge in seven species. There is no *ycf1* in *Brassica juncea*. It is also special in ‘18 R-1’ that *ycf1* is shorter than others and 17,776 bp far from the edge. Near the edge of IRa/LSC, it is *rpl2* in IRa region and trnH in LSC region ranging from 2 bp to 30 bp from boundary (Shi et al. 2020). In some plants, *trnH* is also common in IR region and *rpl22* gene straddles the IRa/LSC boundary (Dan et al. 2019) (Figure 5).

### Cp genome sequence homology analysis

**Figure 6.**
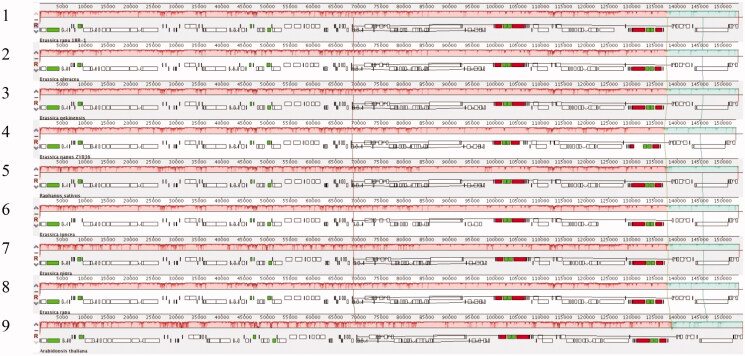
Synteny analysis of chloroplast genomes from nine species in Cruciferae. 1. 18R-1; 2. *Brassica loerance*; 3. *Brassica pekinesis*; 4. *Brassica napus*; 5. *Raphanus sativus*; 6. *Brassica juncea*; 7. *Brassica nigra*; 8. *Brassica rapa*; 9. *Arabidopsis thaliana*.

Using mVISTA online software we assessed the difference of ‘18 R-1’ and other nine *Brassica* species. The results showed that sequences of nine species were highly similar. There was also little variation in the length of each region. Collinearity analysis showed that the cpDNA sequences of nine species did not detect large fragments of gene rearrangement, indicating that the cpDNA sequences were relatively conservative (Figure 6).

### Phylogenetic analysis

**Figure 7.**
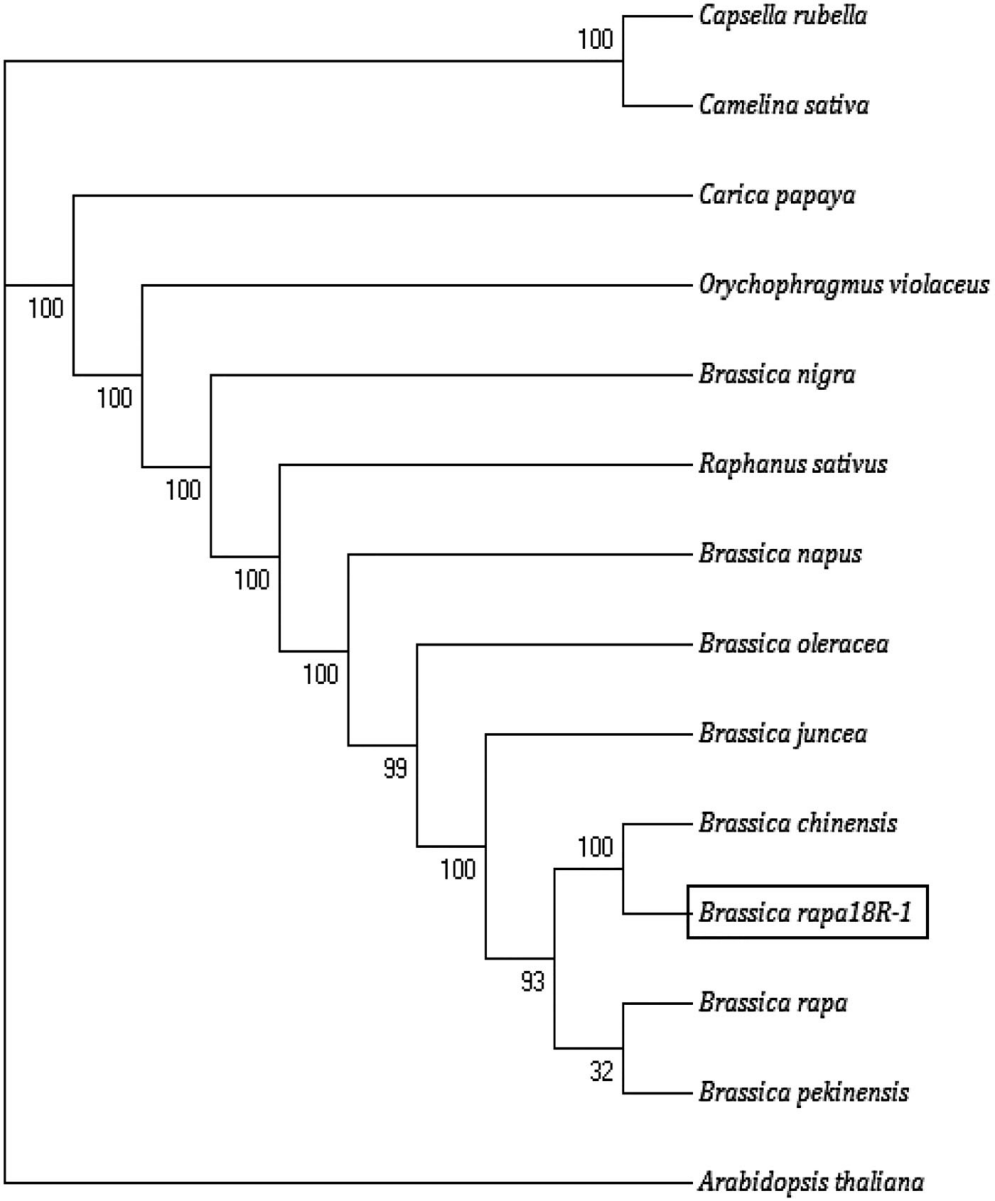
Phylogenetic tree constructed from the maximum likelihood and Bayesian inference based on the complete chloroplast genomes. *Note*: Numbers near each branch is confidence factors in BI.

Phylogenetic analysis was based on the complete cp genome from 14 Cruciferae species (Figure 6). Almost all confidence factors of branches are high (93–100) except for branch between ‘*Brassica rapa*’ and ‘*Brassica pekinesis*’. The higher is the branch’s confidence factor, the more consistent is the guiding value of the evolutionary analysis for the relationship. *Capsella Rubella* and *Camelina sativa* are early differentiated groups. ‘18 R-1’ is a late group. It gathers together with *Brassica chinensis* first, then with *B. rapa* and *Brassica pekinensis*. It means cp genome of ‘18 R-1’ was closely related to *Brassica chinensis*. *Brassica rapa* and *Brassica pekinesis* are located at the innermost of the branch which infers to they are probably the last group of *Brassica* to be differentiated (Figure 7).

## Conclusions

In this study, we reported and analyzed the complete cp genome of a new cultivar ‘18 R-1’ (*B. rapa*), a winter oil rapeseed in China. The cp genome was shown to be more conservative with similar characteristics to other *Brassica* species. An analysis of the phylogenetic relationships among nine species found ‘18 R-1’ was closely related to *B. chinensis.* We can infer that it is different from *B. rapa*. This may be because ‘18-R’ is an oil crop and the cp genome data for *B. rapa* published are from vegetable crops. The results of this study provide an assembly of a whole chloroplast genome of *B. rapa* used as oil crops which might facilitate genetics, breeding, and biological discoveries in the future.

## Data Availability

The authors confirm that the data supporting the findings of this study are available within the article. The accession number on Genebank is MT726210 (https://www.ncbi.nlm.nih.gov/nuccore/MT726210).

## Abbreviations

BI: Bayesian inference
C: Cytosine
cp: Chloroplast
CpSSR: Chloroplast Simple sequence repeats
G: Guanine
IR: Inverted repeat
LSC: Large single copy
NCBI: National center for biotechnology information
RSCU: Relative synonymous codon usage values
SSC: Small single copy
SSRs: Simple sequence repeats
